# Relative importance of dietary uptake and waterborne exposure for a leaf-shredding amphipod exposed to thiacloprid-contaminated leaves

**DOI:** 10.1038/s41598-017-16452-9

**Published:** 2017-11-23

**Authors:** Dominic Englert, Jochen P. Zubrod, Sebastian Pietz, Sonja Stefani, Martin Krauss, Ralf Schulz, Mirco Bundschuh

**Affiliations:** 1 Institute for Environmental Sciences, University of Koblenz-Landau, Landau Campus, Fortstrasse 7, 76829 Landau, Germany; 2 Department Effect-Directed Analysis, Helmholtz Centre for Environmental Research, Permoserstraße 15, 04318 Leipzig, Germany; 3 Department of Aquatic Sciences and Assessment, Swedish University of Agricultural Sciences, Box 7050, 75007 Uppsala, Sweden

## Abstract

Systemic neonicotinoids are commonly used in forest pest management programs. Senescent leaves containing neonicotinoids may, however, fall from treated trees into nearby streams. There, leaf-shredding invertebrates are particularly exposed due to their diet (feeding on neonicotinoid-contaminated leaves) or collaterally via the water phase (leaching of a neonicotinoid from leaves) – a fact not considered during aquatic environmental risk assessment. To unravel the relevance of these pathways we used leaves from trees treated with the neonicotinoid thiacloprid to subject the amphipod shredder *Gammarus fossarum* for 21 days (n = 40) either to dietary, waterborne or a combined (dietary + waterborne) exposure. Dietary exposure caused – relative to the control – similar reductions in gammarids’ leaf consumption (~35%) and lipid content (~20%) as observed for the waterborne exposure pathway (30 and 22%). The effect sizes observed under combined exposure suggested additivity of effects being largely predictable using the reference model “independent action”. Since gammarids accumulated – independent of the exposure pathway – up to 280 ng thiacloprid/g, dietary exposure may also be relevant for predators which prey on *Gammarus*. Consequently, neglecting dietary exposure might underestimate the environmental risk systemic insecticides pose for ecosystem integrity calling for its consideration during the evaluation and registration of chemical stressors.

## Introduction

Neonicotinoids are one of the most widely used insecticides class worldwide^1^. Their tremendous success is due to multiple factors: Firstly, their systemic action facilitates a rapid uptake and distribution in plants and allows for a broad range of application methods thereby reducing the total amount of insecticide needed to be applied^1,2^. Moreover, their broad-spectrum insecticidal properties specifically target insects’ nicotinic acetylcholine receptors while being considerably less toxic for vertebrates^3^. In addition, neonicotinoids replaced insecticides (e.g., organophosphates) that were banned or withdrawn from the market as a result of pest resistance management or increasing regulatory hurdles^1^. Neonicotinoids have, however, raised concerns in the past due to their impact on non-target organisms, in particular pollinators^4–6^. As a consequence, three neonicotinoids (imidacloprid, clothianidin, thiamethoxam) were temporarily banned for certain applications within the European Union^7^. Currently, the European Food Safety Authority is conducting a re-evaluation of the risks these compounds pose for pollinators^8^. Furthermore, the impact of the first neonicotinoid insecticide, i.e., imidacloprid, on aquatic systems has recently been evaluated in the European Union^9^, the United States^10^ and Canada^11^, since neonicotinoids’ frequent use, environmental persistence and physico-chemical properties favour their off-site transport via spray drift, surface runoff^12^ and wastewater discharge^13,14^. Aquatic invertebrate populations and communities are particularly at risk from short-term^15,16^ or chronic^17,18^ neonicotinoid exposure at environmentally relevant levels in surface waters.

Although waterborne exposures are considered the most relevant for neonicotinoids, their systemic nature facilitates a second path that has received little attention in scientific literature and seems ignored during their aquatic environmental risk assessment so far: Dietary exposure through the consumption of neonicotinoid-contaminated plant material. This material may consist of crop post-harvest detritus left on fields^19,20^ as well as senescent leaves falling from neonicotinoid-treated deciduous trees^21,22^ entering adjacent water bodies through lateral movement or vertical fall^23^. In particular leaf-shredding invertebrates (=shredders) – which heavily rely on leaf litter as food source^24^ – might be vulnerable through this pathway. Such dietary exposure might further coincide with exposure via the water phase driven, for instance, by neonicotinoid re-mobilization (i.e., leaching) from leaves into water^21,22^. The relative importance of these exposure pathways (dietary vs. waterborne) has, however, not yet been disentangled, though the significance of dietary exposure under a combined exposure scenario has been suggested in an earlier publication^25^.

Therefore, the present study aimed to unravel the relevance of these pathways by subjecting the shredder *Gammarus fossarum* (Koch) – an amphipod frequently used in non-standard aquatic toxicity studies^26^– for 21 days to leaves from neonicotinoid-treated black alder trees, using thiacloprid (THI) as a model substance. These leaves served as the only neonicotinoid source: gammarids either faced dietary exposure – i.e., feeding on THI-contaminated leaves while a flow-through system prevented THI from accumulating in the water phase – waterborne exposure (through leaching of THI from leaves) or combined exposure (i.e., dietary + waterborne). Besides the test organisms’ survival, leaf consumption and THI body burden, gammarids’ body weight and lipid content were determined as a proxy for their energy reserves and physiological fitness^27^. Based on our previous work^25^, we expected the most severe effects in the combined exposure scenario, while dietary exposure alone was hypothesized to be less or equally important as waterborne exposure. In case the effects induced by combined exposure turned out to exceed the effect sizes induced by each individual exposure pathway, we further assumed that they could be predicted – although consisting of two exposure pathways instead of a mixture of different chemicals – by one of the most commonly used reference models, namely “independent action” (IA)^28^. Thereby, the present work assessed the relevance of a pathway – i.e., dietary exposure – that is not well reflected in current aquatic risk assessment of neonicotinoids or systemic pesticides in general.

## Results

### THI concentrations in leaves and water

**Figure 1:**
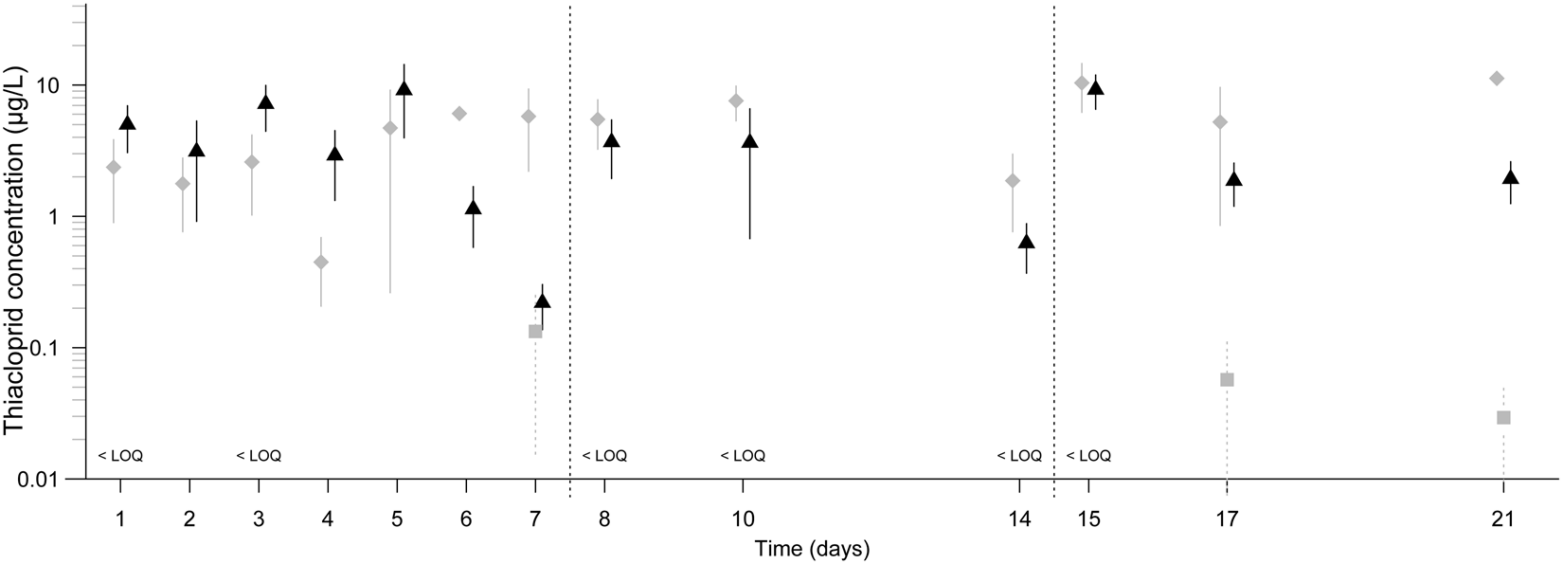
THI water concentrations (mean ± SE; n = 3–4) measured during the 21-day feeding experiment.  marks concentrations of the waterborne,  the dietary and ▲ the combined exposure treatment, respectively. Except for three cases, THI water concentrations in the dietary exposure treatment were below the LOQ (0.01 µg/L).

THI residues in leaves quantified prior to the feeding experiment were 245.3 ± 6.9 µg/g (mean ± standard error (SE); n = 3). During the first, second and third week, leaching of THI from leaves into water resulted in mean water concentrations of 3.4, 5.0 and 9.0 µg THI/L in the waterborne exposure scenario. Similarly, weekly mean water concentrations of 4.1, 2.7 and 4.3 µg THI/L were measured in the combined exposure scenario (Fig. 1). In contrast, THI water concentrations in the dietary exposure scenario barely exceeded the limit of quantification (LOQ = 0.01 µg/L; Fig. 1).

### Survival of *G. fossarum*

**Figure 2:**
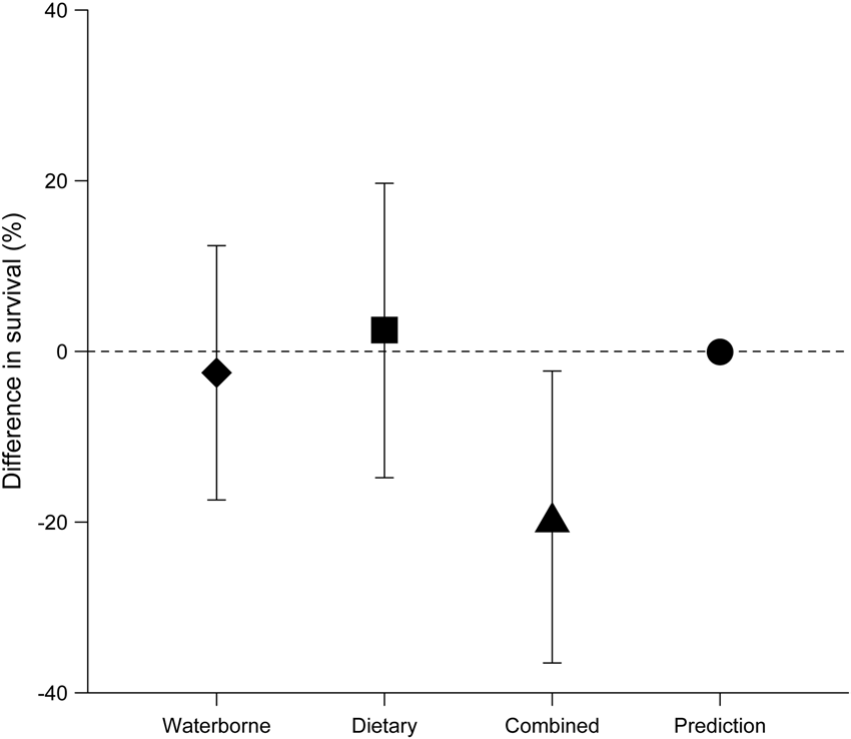
Mean difference in survival (±95% CIs) of *G. fossarum* (after 21 days; n = 40) exposed towards THI via different pathways. The prediction of the IA model is also displayed as a point estimate (●).

After 21 days, survival of *G. fossarum* experiencing waterborne (difference in proportions: −2.5%; Chi-squared test: p = 1) or dietary THI exposure (difference in proportions: 2.5%; Chi-squared test: p = 1; Fig. 2) deviated only marginally from the corresponding control. In the combined exposure scenario, a 20% reduced survival (compared to the corresponding control; Chi-squared test: p = 0.15; Fig. 2) was observed, which deviated significantly from the IA model prediction, which suggested an effect size of approximately zero.

### Gammarids’ leaf consumption

**Figure 3:**
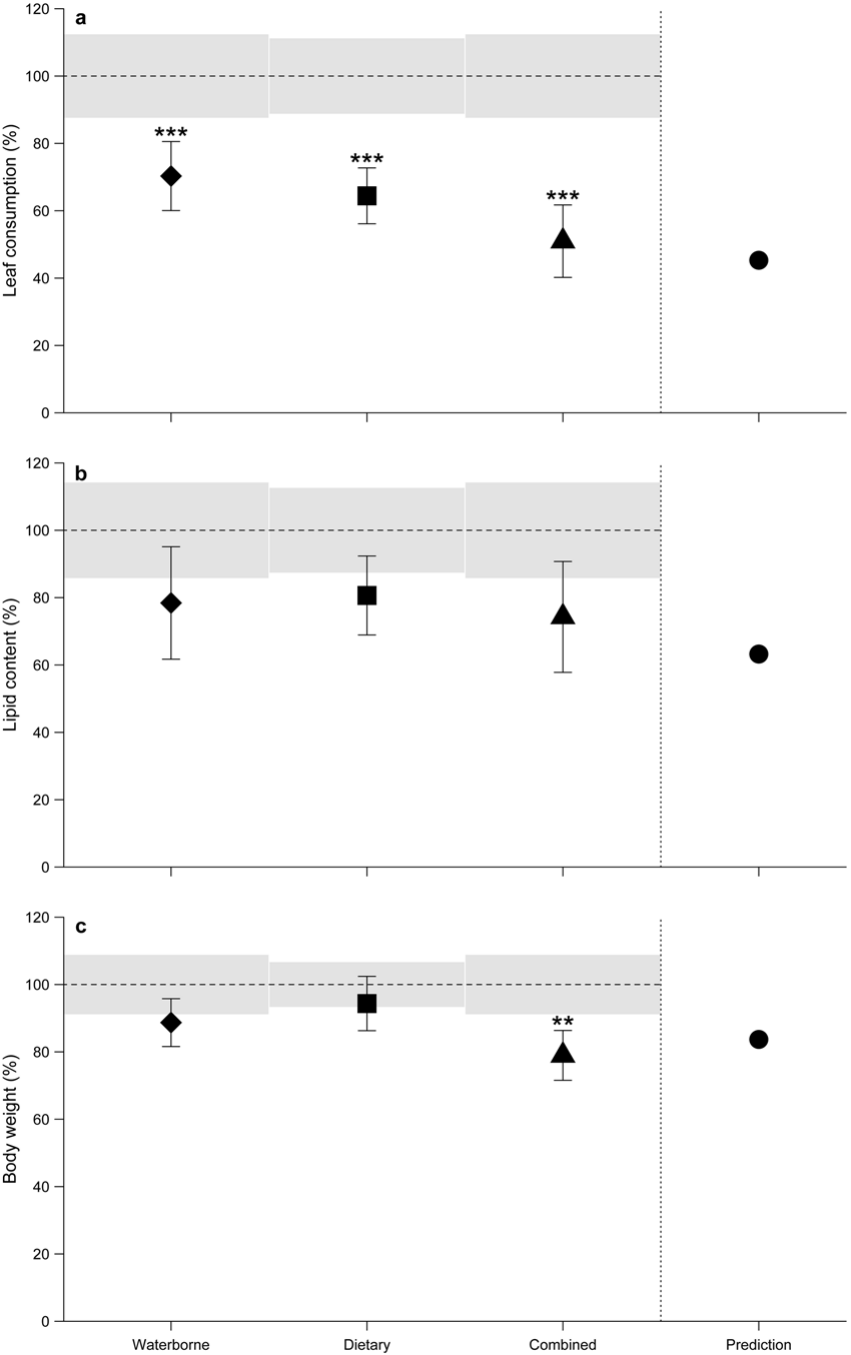
Mean (±95% CIs) (**a**) leaf consumption, (**b**) lipid content and (**c**) body weight of gammarids exposed for 21 days to thiacloprid, relative to the corresponding control (dashed line). Grey areas indicate the 95% CIs of the corresponding control. Please note that the dietary exposure treatment was compared to a separate control due to the flow-through system used (see *Methods* section). The predictions derived from IA models are also indicated as point estimate (●). Asterisks denote significant differences compared to the respective control, *p* < 0.01 (**), *p* < 0.001 (***).

The cumulative leaf consumption of *G. fossarum* was significantly reduced by waterborne (by 30%; *t*-test: p < 0.001; n = 33/34) and dietary (by 36%; *t*-test: p < 0.001; n = 32/33; Fig. 3a) THI exposure relative to the corresponding control. Based on these results, the IA model predicted a 55% reduction in leaf consumption for the combined exposure, which corresponded well with the observed and statistically significant reduction of 49% (*t*-test: p < 0.001; n = 26/34; Fig. 3a).

### Gammarids’ lipid content & body weight

For gammarids exposed via the water phase, a non-significant decrease in lipid content and body weight (by 22 and 11%, respectively; *t*-test: p = 0.054/0.098; n = 21–22/29–30; Fig. 3b,c) was observed when compared to the control. The dietary exposure pathway decreased *G. fossarum’*s lipid content near-significantly by 19% (*t*-test: p = 0.054; n = 24/25), whereas animals’ body weight was only slightly and non-significantly reduced (effect size: 6%; Wilcoxon rank-sum test: p = 0.263; n = 28/32; Fig. 3b,c). The by 26 and 21% reduced lipid content and body weight (only statistically significant for the latter; *t*-test: p = 0.054/0.003; n = 21–22/22–30; Fig. 3b,c) of gammarids in the combined exposure treatment deviated slightly from the effect sizes predicted by the IA model (37 and 16%) but were still within the 95% confidence interval (CI) range.

### Gammarids’ THI body burden

**Figure 4:**
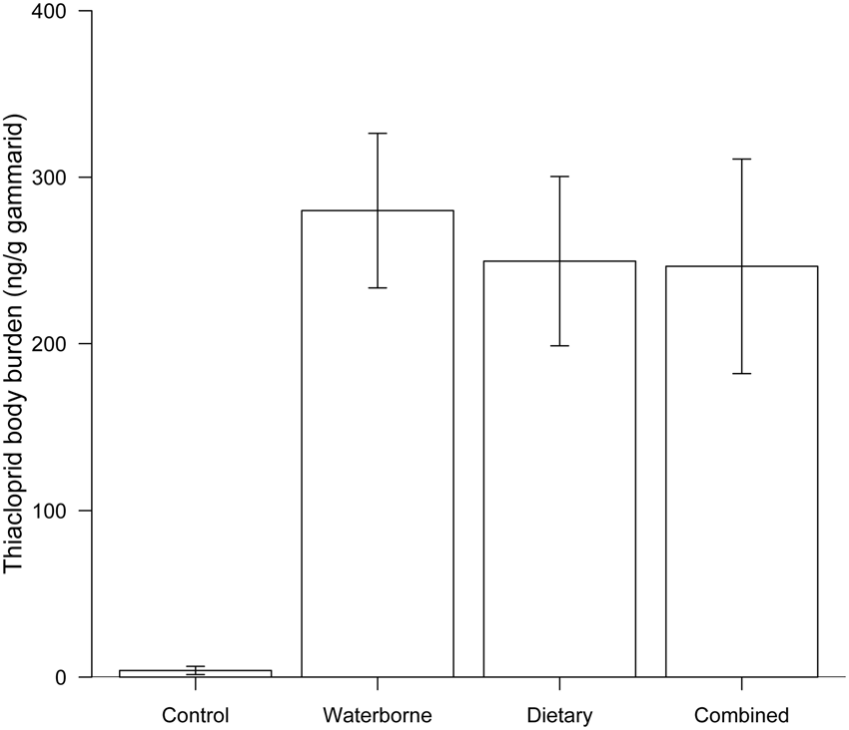
Mean THI body burden (±SE) in gammarids. Residues were measured (n = 4–5) after 21 days of THI exposure via water, diet or a combination of both.

THI was detected in two out of five gammarids in the control treatment at levels marginally above the LOQ (1 ng/g), namely 9 and 11 ng THI/g wet weight. In contrast, animals exposed to THI for 21 days via the water phase, their diet or both displayed body burdens that exceeded the maximum residue found in gammarids of the control group by up to 25-fold, namely 279.9 ± 46.4, 249.6 ± 50.8 and 246.5 ± 64.5 ng THI/g gammarid. Residue levels did not differ significantly between the three exposure scenarios (Tukey-test: p ≥ 0.90; n = 4–5; Fig. 4).

## Discussion

The dietary pathway significantly reduced the leaf consumption (by 36%) and, though non-significant, the lipid content (by 19%) of *G. fossarum* to an extent comparable to effects observed under waterborne exposure (Fig. 3a,b) while gammarids’ body weight was only marginally altered (Fig. 3c). As most research regarding neonicotinoids’ effects on aquatic systems focused exclusively on waterborne exposure pathways (e.g., see those reviewed in Morrissey *et al*.^29^), studies solely examining dietary and thus excluding waterborne pathways are lacking. Therefore, the present study is, to the best of our knowledge, the first addressing this challenge by means of a flow-through system, which kept THI water concentrations in the dietary exposure scenario below or only slightly above detectable levels (i.e., LOQ: 0.01 µg/L; Fig. 1). While the leaves’ THI concentrations presumably declined throughout each 7-day exposure period^21^, aqueous THI concentrations might have temporarily been above the LOQ, particularly near the leaves’ surface. The measured THI water concentrations, nonetheless, strongly suggest that the major share of the effects observed in the dietary exposure scenario was delivered via the consumption of THI-contaminated leaves.

Neither the waterborne exposure nor the dietary uptake of THI-contaminated leaves did, however, affect the survival of *G. fossarum* (Fig. 2). Whereas scientific literature lacks information regarding dietary neonicotinoid exposure on gammarids’ survival, results observed in the waterborne exposure scenario of the present study (Fig. 2) are in accordance with former publications. Firstly, the weekly average THI water concentrations (Fig. 1) were, although highly variable presumably due to unequal spatial distribution of neonicotinoids in tree foliage^30,31^, up to 95-fold below the 96-h median lethal concentration (i.e., LC_50_: 320 µg THI/L) reported for *G. pulex* ^32^. Moreover, in one of our previous studies we observed no THI related mortality in *G. fossarum* after 7 days of waterborne exposure towards 16 µg/L as well as an 7-day LC_20_ of 33 µg THI/L under a combined exposure szenario^25^.

In the present study, both the dietary and waterborne exposure pathway induced a reduction in gammarids’ leaf consumption (by 36 and 30%, respectively; Fig. 3a), a sublethal response regularly reported in literature as a consequence of exposure towards THI^25,33–35^ or other neonicotinoids^17,25,36^. This reduced energy uptake (i.e., in the form of leaves; Fig. 3a) eventually caused the observed reduction (up to 22%) in *G. fossarums’* lipid reserves (Fig. 3b), a pattern also reported for *G. pulex* in response to a 21-day imidacloprid exposure^17^. Additionally, the allocation of energy to detoxification processes^37^ or cellular repair processes (i.e., to counteract lipid peroxidation due to neonicotinoid induced oxidative stress; e.g., as shown for *G. fossarum* ^38^) might have contributed to the reduction in gammarids’ lipid reserves (Fig. 3b).

While dietary as well as waterborne THI exposure caused comparable effects in *G. fossarum* (i.e., regarding survival, leaf consumption, lipid content and body weight; Figs 2 and 3a–c), those induced by the combined exposure scenario exceeded in their magnitude – independent of the variable – those observed for each of the exposure pathways individually (Figs 2 and 3a–c). Only gammarids’ THI body burdens were at comparable levels irrespective of the exposure pathway (except for control animals; Fig. 4). Since THI body burdens in another amphipod species from a pesticide-impacted river, namely *Dikerogammarus* sp., did not exceed 0.39 ng THI/g^39^, the concentrations found in two of our control animals (i.e., 9 and 11 ng THI/g) are likely attributed to minor laboratory cross-contamination after the termination of the experiment and not field-exposure preceding our study. Although neonicotinoids – as is expected based on their log P_ow_ (between −0.66 and 1.26)^40^ – supposedly do not accumulate in organisms’ tissues except for the nervous system (due to the insecticides’ affinity for the nicotinic receptors), they are taken up to an increasing extent with rising aqueous neonicotinoid concentration and exposure time^41^. After a few days of exposure, however, the uptake of THI into *G. fossarum* might plateau – as reported for imidacloprid in the mayfly nymph *Isonychia bicolor* after ~5 days^42^ – which likely explains the absence of any differences in gammarids’ THI residue levels at the termination of our 21-day study (Fig. 4). Despite the comparable THI residue levels detected in gammarids exposed via the water phase, their diet or both (Fig. 4), the location where the neonicotinoid is accumulated and, therefore, possibly the affected target site might differ depending on the exposure pathway. This may not only be relevant for neonicotinoids’ trophic transfer to predators engulfing or merely piercing their prey^43^ but could also explain the mostly additive effects observed under combined exposure (Figs 2 and 3a–c). The latter have largely been predictable in their magnitude by the IA model (for the variables leaf consumption, lipid content and body weight; see Fig. 3a–c), which assumes different molecular target sites to be affected^44^. The only exception to this good conformity between predictions and observations was gammarids’ survival, which was substantially underestimated by the model (Fig. 2). This deviation indicates a synergistic effect on gammarids’ survival triggered by the combined exposure via both pathways, though the underlying mechanism remains unclear. Given the potential for additive or synergistic effects under a combined exposure scenario, as well as the adverse effects we observed in gammarids under dietary exposure alone (Fig. 3a,b), the risk posed by neonicotinoid-contaminated leaves falling into streams needs further consideration.

The vast number of studies reporting the introduction of neonicotinoids to surface waters via wastewater discharge^14^, agricultural spray drift and surface runoff from crops^12^, as well as the numerous exceedances of regulatory acceptable concentrations^45^ emphasize the relevance of waterborne neonicotinoid exposure for aquatic organisms. But also relatively pristine streams may receive neonicotinoids following application as part of forest pest management programs^46^. Although the injection of neonicotinoids directly into the tree trunk may, in contrast to their soil application, limit the initial distribution of the insecticide within the environment^47^, leaves from neonicotinoid-treated trees might still, regardless of the pesticides’ application method, be transported into nearby streams. There the relatively fast re-mobilization of leaf-associated neonicotinoids into water^21^ may presumably limit the relevance of the dietary exposure to a few days after the leaves’ introduction into the stream^22^. Moreover, depending on the neonicotinoid compound and the shredders’ ability to detect contaminated food, dietary exposure might be avoided if an uncontaminated alternative is available^25^. Although autumn leaf fall might represent a peak input event for neonicotinoid-contaminated leaves, only low aqueous concentrations (and hence low waterborne exposure) might be expected in streams providing sufficient dilution^22^. However, when the stream receiving contaminated leaves fails to provide sufficient dilution as a consequence of low discharge, organisms might be adversely affected through exposure via the water phase and their diet at the same time (cf. Figure 3a,b).

Irrespective of the pathway investigated in the present study, neonicotinoid exposure at environmentally relevant levels (regarding both water^29,48^ and leaves^22^) triggered adverse effects in *G. fossarum* (Fig. 3a,b) that may impair leaf litter breakdown and eventually energy transfer processes in heterotrophic streams^49,50^. Although the susceptibility of *G. fossarum* towards THI may be even higher in other populations^34^, reduced leaf consumption may lower their feces production, restricting the amount of food available for collectors including juvenile gammarids^24,51^. Furthermore, it could be hypothesized that the consumption of contaminated leaves by shredders would result in the excretion of feces similarly contaminated representing a potential concern for collecting and filtering invertebrates of local or downstream communities^52^.

In addition, as lipids and body weight are indicative for recourses organisms can invest into reproduction, there may be implications for gammarids’ population development^53,54^. Reduced reproduction would eventually result in a lower abundance of this keystone species^55^ and in turn further reduce their contribution to local leaf litter breakdown. Vertebrate and invertebrate predator populations, which frequently feed upon *Gammarus* ^56^, may also be adversely affected by the lowered prey abundance or through the consumption of neonicotinoid-contaminated prey^57^.

In the European Union, testing for dietary effects is only recommended for substance characterized by extremely high octanol/water partition coefficients (log P_ow_ > 6)^58^, while for systemic insecticides (normally characterized by high hydrophilicity and low log P_ow_) this pathway is considered irrelevant. Results of the present study, however, underpin the relevance of dietary exposure pathways for this group of insecticides (as well as for other pollutants^59^) – for aquatic shredders in particular. Considering that the control of native and invasive pests in forests becomes – under global climate change predictions – increasingly relevant^60^, the input of neonicotinoid-contaminated leaves into surface waters (e.g., during autumn leaf fall) should not be ignored as an exposure pathway during their aquatic environmental risk assessment.

## Methods

### Source of plant material, neonicotinoid application and preparation of leaf discs

The procedure used to generate THI-free and THI-contaminated black alder leaves for the present study is described in detail in Englert *et al*.^22^. Briefly, black alder trees were soil drenched in June 2014 with either 500 mL tap water or 500 mL tap water containing the neonicotinoid product Calypso^®^ (40% THI; Bayer CropScience GmbH, Langenfeld, Germany; dose: 0.6 g THI/cm trunk diameter at breast height). Leaves were collected in October 2014, shortly before leaf fall, and stored frozen at −20 °C until further use. Leaf discs (diameter = 1.0 cm) were cut from leaves using a cork borer, freeze-dried and subsequently weighed to the nearest 0.01 mg to determine their initial dry weight.

### Test organisms

As described in Bundschuh *et al*.^61^, adult *G. fossarum* of 6–8 mm body length and visibly free of macro-parasites were collected one week prior to the start of the experiments from the stream Hainbach (49°14′N; 8°03′E). The stream is located in the Palatinate forest upstream of any settlement and agricultural activity and the surrounding forest has no history of neonicotinoid use. Pre-exposure of gammarids towards neonicotinoids is therefore likely negligible. Since the present study was conducted during gammarids’ reproductive rest (October to November)^62^, organisms could not be separated by sex. Consequently, both male and female gammarids were used. This procedure might increase the variability in the endpoints investigated but also the study’s relevance for effects at the population level. Seven days prior to the start of the bioassay, organisms were kept in aerated stream water at 16 ± 1 °C, fed *ad libitum* with black alder leaves and gradually adapted to SAM-S5 medium^63^ (=test medium).

### Bioassay design

**Figure 5:**
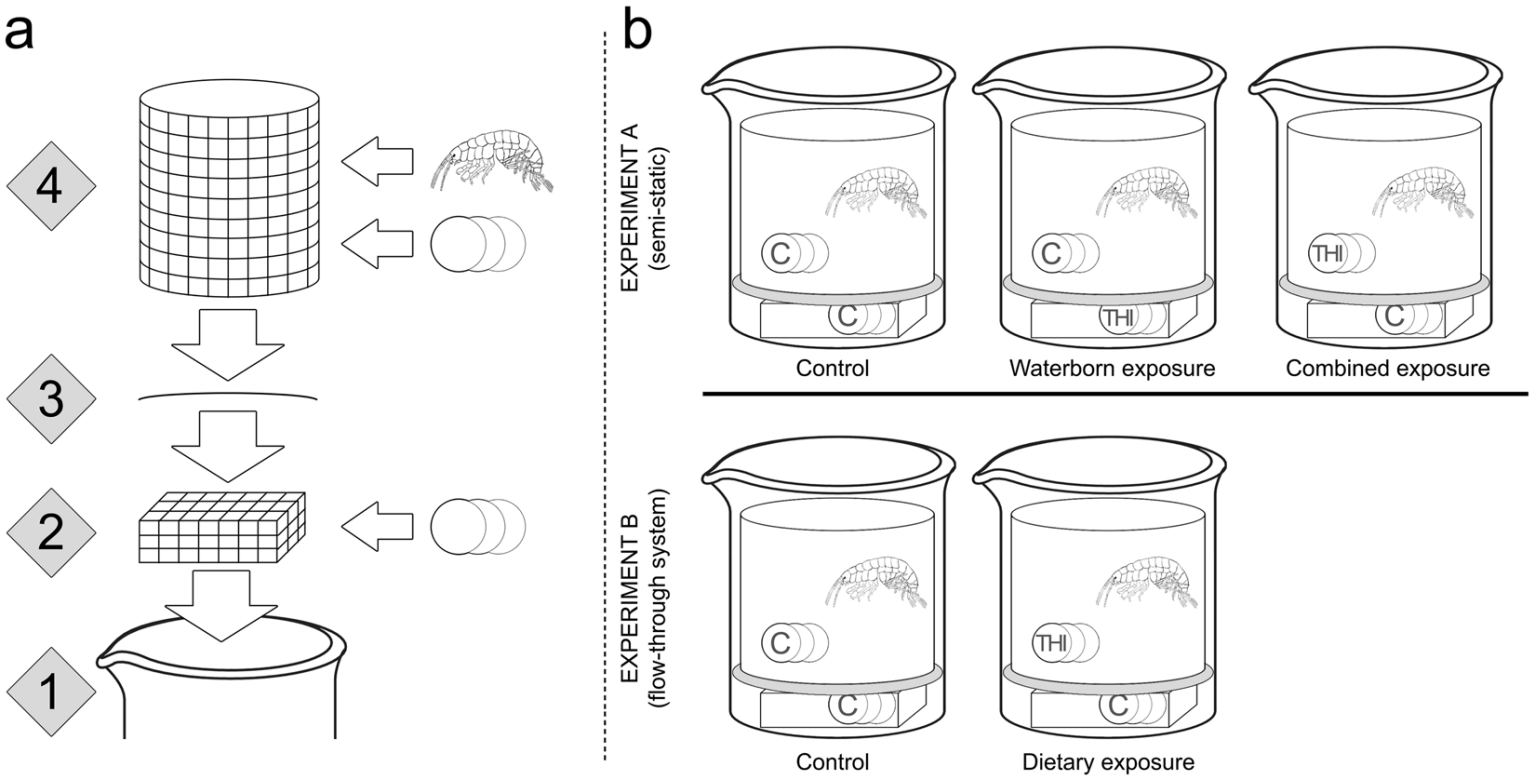
Scheme illustrating the setup of the bioassay. (**a**) At the bottom of a (1) 250-mL glass beaker filled with 200 mL test medium a (2) cuboid shape cage, containing three leaf discs, was situated. A (3) watch glass placed on top of the cuboid cage separated the latter from a cylindrical shape cage containing three leaf discs as well as one *G. fossarum*. (**b**) The different exposure scenarios were realized by manipulating the position of THI-free (C) and THI-contaminated (THI) leaf discs in these cages. While the waterborne and combined exposure scenario used a semi-static regime, the dietary exposure scenario was realized using a flow-through system that kept THI water concentrations at negligible levels.

Each replicate consisted of a 250 mL glass beaker filled with 200 mL test medium. Each beaker was equipped with two cages made of stainless steel mesh (mesh size: 0.5 mm) – a cuboid shape cage at the bottom (4.0 cm × 4.0 cm × 0.5 cm) and a cylindrical shape cage above (height: 8.0 cm, diameter: 5.5 cm) – that were separated by a watch glass (diameter: 6.0 cm; Fig. 5a)^64^. The bottom cage contained three leaf discs that were protected from organisms’ feeding and allowed controlling for abiotic and microbial leaf mass loss. The upper cage contained one *G. fossarum* and three discs cut from black alder leaves as food. The different exposure scenarios were realized by manipulating the position of THI-free and THI-contaminated leaf discs in these cages as follows: For the control treatment, THI-free leaf discs were placed in both the upper and the bottom cage. Gammarids in the waterborne exposure treatment were allowed to feed on the THI-free leaf discs placed in the upper cage, while THI gradually leached from THI-contaminated leaf discs, which were placed in the bottom cage. For the combined exposure treatment, in contrast, THI-free leaf discs were placed in the bottom cage while in the upper cage THI-contaminated leaf discs served as food for *Gammarus*. As Kreutzweiser *et al*.^21^ reported only a marginal accumulation of another neonicotinoid (i.e. imidacloprid) with similar physico-chemical properties as THI in leaf material when applied via the water phase, the diet-related uptake of THI during the waterborne exposure treatment is considered negligible in the present study. The dietary exposure treatment was conducted – with THI-contaminated discs in the upper cage and THI-free discs in the bottom cage – using a flow-through system, which continuously (~45 times/d) renewed the test medium and kept THI water concentrations at negligible levels (i.e., below the LOQ; Fig. 1). A separate control treatment, accounting for any potential effects of the water renewal process itself, was also set up. Each of these five treatments was replicated 40 times. A scheme of the experimental design is illustrated in Fig. 5b.

Beakers were placed in a climate controlled chamber at 16 ± 1 °C in total darkness and aerated throughout the 21 day study duration. At weekly intervals, leaf discs in every treatment were renewed together with the test medium to maintain a continuous THI exposure. Thereby, cages allowed a gentle transfer of the test organisms to new vessels containing fresh test medium and leaf discs. Remaining leaf discs and any leaf tissue shredded off were removed from glass beakers, freeze-dried separately and weighed to the nearest 0.01 mg. At the same time, survival of gammarids was monitored. Gammarids were considered dead if no movement was observed after several gentle touches with the tip of a glass pipette. 10-mL water samples were taken daily (n = 4) during the first week as well as on the first, third and last day (n = 3) during following two weeks. Samples were stored at −20 °C until further use. At the termination of the experiment, gammarids were transferred to fresh test medium for 1 h to remove THI residues possibly adsorbed to their bodies’ surface. Subsequently, test organisms were carefully blotted dry with a clean tissue, shock frozen in liquid nitrogen and stored in individual glass tubes at −75 °C until further use.

### Thiacloprid quantification in leaves, water and gammarids

THI was extracted from alder leaves using an ASE™ 350 Accelerated Solvent Extractor system (Thermo Scientific™ Dionex™, Sunnyvale, CA; USA)^22^. Separation of THI from leaf extracts and water samples was done with an ultrahigh performance liquid chromatography–mass spectrometry system equipped with an EQuan MAX system, while for quantification, a single quadrupole mass spectrometer equipped with an electrospray ionization source was used^22^. THI was identified at the accurate ion mass of m/z = 253.0309. External calibration with matrix-matched standards (prepared out of blank leaf extracts or test medium) was used. The limits of quantification (LOQ) and the limits of detection (LOD) for THI in leaf samples were 0.11 and 0.03 µg/g. For THI measured in water samples, the LOQ was defined as the lowest calibration level (i.e., 0.01 µg/L) due to the absence of signals in matrix-matched blank samples^65^.

THI was extracted from gammarids using the method of Inostroza *et al*.^39^. In brief, frozen gammarids were transferred into a 10 mL centrifuge tube, 1 mL of LC-MS grade acetonitrile, 1 mL of LC-MS grade water and 0.5 mL of LC grade hexane were added and the sample was homogenized using an UltraTurrax. Phase separation between water and acetonitrile was induced by addition of 400 mg of MgSO_4_ and 100 mg of NaCl. The hexane phase was removed and the acetonitrile phase transferred into a 2 mL glass vial, evaporated to dryness and reconstituted in 500 µL of methanol:water (70:30). THI was analysed using liquid chromatography-high resolution mass spectrometry (Ultimate 2000 LC system coupled to a QExactive Plus MS via a heated electrospray ionisation source, all from Thermo Scientific™). Separation was conducted using a methanol:water gradient (both eluents containing 0.1% of formic acid) on a Kinetex C18 EVO column (50 × 2.1 mm, 2.6 µm particle size, Phenomenex). THI was measured in full scan mode and quantified using the accurate mass of m/z = 253.0309. Method matched calibration was employed (i.e., spiked solvents were processed the same way as the gammarid samples) using imidacloprid-d4 as internal standard. The LOQ was 1 ng/g wet weight.

### Quantification of gammarids’ body weight and lipid content

For the determination of gammarids’ body weight, animals were freeze-dried (for 24 h) and weighed to the nearest 0.01 mg. Subsequently, the lipid content of gammarids (n = 21–25) was analysed as described by Van Handel^66^ and modified by Zubrod *et al*.^64^ for use with a microplate reader (Tecan Infinite M200, Tecan Group, Crailsheim, Germany). After extraction with 1:1 chloroform:methanol (v:v), lipids reacted with sulfuric acid and vanillin-phosphoric acid reagent. For quantification of the lipid content, absorbance at 490 nm was measured and read against a standard curve prepared from commercially available soybean oil (Sojola Soja-Öl, Vandemoortele, Herford, Germany). Lipid content was finally normalized to gammarid dry weight (µg/mg gammarid).

### Calculations and statistics

Gammarids’ consumption of alder leaf discs (in mg leaf/animal/day; corrected for the abiotic and microbial leaf mass loss as determined from the bottom cages) was calculated for each week as described in Bundschuh *et al*.^61^, neglecting the animals dry weight, as this was only measured at the termination of the experiment and would thus induce additional uncertainties. Moreover, the cumulative consumption (in mg leaf/animal/day) was calculated over the entire study duration (i.e., 21 day). In the remainder of this publication the term “leaf consumption” refers – if not indicated otherwise – exclusively to cumulative consumption. Replicates in which gammarids managed to escape their cage or died were discarded from statistical analyses (except for survival).

Gammarids’ cumulative leaf consumption, lipid content and body weight (as dry weight) were checked for normality by visual inspection and Shapiro-Wilk’s test, while homoscedasticity was tested using Bartlett’s test. To test for statistically significant differences relative to the corresponding control, Student’s *t*-tests or, if assumptions for parametric testing were violated, non-parametric Wilcoxon rank-sum tests were conducted. Although our experimental design consisted of two separate experiments, formally rendering any alpha level adjustment for the control vs. dietary comparison unnecessary, alpha-corrections for three comparisons were applied in all cases using Bonferroni-Holm-adjustment. Since the interpretation of our data is mainly driven by effect sizes, this rather conservative approach further reduced type I errors, however, this hardly affected any drawn conclusions. Gammarids’ survival was compared to the corresponding control using Chi-squared tests.

Moreover, results observed in the combined exposure treatment were tested for compliance with the reference model “independent action” (IA)^28^. IA was calculated by multiplying the average mortality, leaf consumption, body weight or lipid content (as proportion of untreated controls) observed in the single exposure treatments. Although IA was originally designed for binominal responses (e.g., alive/dead) and probabilities, it can be used with gradual data that do not meet the theoretical assumptions of IA^67^.

Gammarids’ THI body burdens were checked for normality and homoscedasticity as described above while analyses of variance followed by Tukey-test was used to test for statistically significant differences between the waterborne, dietary and combined exposure treatment. All null hypothesis significance tests were supplemented by 95% CIs^68^ and are given in Supplementary Table S1. For all statistics and figures, R version 3.1.1 for Mac was used. The datasets generated during and/or analysed during the current study are available from the corresponding author on reasonable request.

## Electronic supplementary material

Supplementary Information
