# Supplementary material for: Relative importance of dietary uptake and waterborne exposure for a leaf-shredding amphipod exposed to thiacloprid-contaminated leaves
Source: Sci Rep. 2017 Nov 23;7:16182. doi: 10.1038/s41598-017-16452-9 (PMC5700932; doi:10.1038/s41598-017-16452-9)
Supplement: Supplementary file 1 — Supplementary Information [file 41598_2017_16452_MOESM1_ESM.pdf]

Supplementary Information for the paper:

Relative importance of dietary uptake and waterborne exposure for a leaf-shredding amphipod exposed to thiacloprid-contaminated leaves

Authors: Dominic Englert[1], Jochen P. Zubrod[1], Sebastian Pietz[1], Sonja Stefani[1], Martin Krauss[2], Ralf Schulz[1] & Mirco Bundschuh[3]

[1]Institute for Environmental Sciences, University of Koblenz-Landau, Landau Campus, Fortstrasse 7, 76829 Landau, Germany

[2]Department Effect-Directed Analysis, Helmholtz Centre for Environmental Research, Permoserstraße 15, 04318 Leipzig, Germany

[3]Department of Aquatic Sciences and Assessment, Swedish University of Agricultural Sciences, Box 7050, 75007 Uppsala, Sweden

Supplementary Table S1. Means and corresponding 95% confidence intervals (CIs) of *G. fossarums'* survival, leaf consumption, lipid content and body weight observed in the respective treatments.

Endpoint	Treatment	Mean	95%CIs
Survival	Control	90.0%	76.9 to 96.0
	Waterborne	87.5%	73.9 to 94.5
	Combined	70.0%	54.6 to 81.9
	Control	80.0%	65.2 to 89.5
	Dietary	82.5%	68.1 to 91.3
Leaf consumption	Control	0.42 mg/animal/d	0.37 to 0.47
	Waterborne	0.30 mg/animal/d	0.25 to 0.34
	Combined	0.21 mg/animal/d	0.17 to 0.26
	Control	0.51 mg/animal/d	0.45 to 0.57
	Dietary	0.33 mg/animal/d	0.29 to 0.37
Lipid content	Control	119.86 µg/mg	102.67 to 137.05
	Waterborne	94.00 µg/mg	73.99 to 114.01
	Combined	89.02 µg/mg	69.29 to 108.76
	Control	131.62 µg/mg	114.84 to 148.39
	Dietary	106.14 µg/mg	90.72 to 121.57
Body weight	Control	3.55 mg	3.32 to 3.86
	Waterborne	3.14 mg	2.89 to 3.40
	Combined	2.80 mg	2.54 to 3.06
	Control	3.68 mg	3.43 to 3.93
	Dietary	3.47 mg	3.18 to 3.77
